# Quercetin Attenuates Iron Overload-Induced Renal Injury via Activating Nrf2/xCT/GPX4 Signaling to Inhibit Ferroptosis

**DOI:** 10.3390/life16030372

**Published:** 2026-02-25

**Authors:** Xiaoyi Wang, Wenmi Li, Wenzheng Yuan, Ziyu Wei, Zixuan Yang, Zichun Zhang, Zhibin Sun, Guojie Ji, Huanhuan Hu

**Affiliations:** 1 Key Laboratory of Fertility Preservation, School of Life Sciences and Technologies, North Henan Medical University, West Section of Changjiang Road, Pingyuan Demonstration Area, Xinxiang 453003, China; 2 Key Laboratory of Biological Resources and Ecology of Pamirs Plateau in Xinjiang Uygur Autonomous Region, College of Life and Geographic Sciences, Kashi University, Kashi 844000, China; 3 Department of Biomedical Engineering, Nanjing University of Aeronautics and Astronautics, Nanjing 211106, China

**Keywords:** quercetin, kidney injury, iron overload, NRF2/xCT/GPX4, ferroptosis

## Abstract

Iron overload, a key driver of ferroptosis, results from excessive iron accumulation in tissues and contributes to organ injury, including renal dysfunction. Increasing evidence indicates that ferroptosis plays an important role in the pathogenesis of kidney diseases. Natural antioxidants capable of regulating ferroptosis have therefore attracted growing attention. Quercetin (Que), a naturally occurring flavonoid, possesses well-documented antioxidant and anti-inflammatory properties and may provide protection against iron overload-induced renal injury. Present study aimed to clarify the molecular mechanisms underlying iron overload-induced nephrotoxicity and to evaluate the protective effects of Que through modulation of ferroptosis-related signaling pathways. Using in vivo and in vitro experimental approaches, we found that Que markedly reduced oxidative stress by regulating reactive oxygen species (ROS) levels, intracellular iron homeostasis, and the expression of ferroptosis-related proteins in renal tissues and HK-2 cells. The results demonstrate that iron overload induces renal injury primarily through activation of ferroptosis, characterized by iron-dependent lipid peroxidation and subsequent cellular damage. Importantly, Que significantly attenuated iron overload-induced renal injury by activating the NRF2/SLC7A11 (xCT)/GPX4 signaling pathway, thereby restoring antioxidant capacity and inhibiting ferroptotic cell death. In conclusion, Que protects against iron overload-induced renal injury by enhancing antioxidant defenses and maintaining iron homeostasis through inhibition of ferroptosis. These findings suggest that Que may represent a potential therapeutic strategy for kidney diseases associated with iron overload.

## 1. Introduction

Iron is an essential trace element that plays pivotal roles in oxygen transport and cellular metabolism [1]. However, iron accumulation beyond physiological thresholds leads to iron overload, which promotes the generation of reactive oxygen species (ROS) and triggers ferroptosis, a regulated form of cell death characterized by lipid peroxidation [2,3]. Excessive ROS production, depletion of glutathione (GSH), and impaired glutathione peroxidase 4 (GPX4) activity are key drivers of iron-induced nephrotoxicity. Disruption of redox homeostasis consequently leads to renal tubular epithelial cell damage, inflammation, and ultimately chronic kidney disease (CKD) [4,5]. Therefore, maintaining systemic iron homeostasis is essential for preserving cellular integrity and normal physiological function [6,7].

The kidneys play a central role in systemic iron regulation through iron reabsorption, utilization, and metabolic control. However, dysregulated iron metabolism renders renal tissues particularly susceptible to iron overload and ferroptosis-associated injury [8]. Increasing evidence identifies ferroptosis as a key pathological mechanism in iron-induced kidney damage [9]. Nevertheless, the molecular pathways linking iron overload to renal ferroptosis remain incompletely understood.

Ferroptosis research has expanded rapidly in recent years. As a distinct iron-dependent form of regulated cell death, ferroptosis is controlled by multiple metabolic processes, including redox homeostasis, iron metabolism, mitochondrial function, and amino acid, lipid, and glucose metabolism [10,11]. Iron overload, a defining feature of ferroptosis, promotes excessive iron deposition in multiple organs, leading to tissue injury and functional impairment [12]. Current evidence suggests that iron overload-induced ferroptosis contributes to kidney disease through several interconnected mechanisms. Disrupted redox balance results in ROS accumulation, GSH depletion, and GPX4 inactivation, ultimately promoting lipid peroxidation and renal injury [13]. Elevated ROS levels also damage subcellular structures such as mitochondria and the endoplasmic reticulum in renal tubular epithelial cells, while activating pro-inflammatory pathways including nuclear factor-κB (NF-κB) signaling and the NOD-like receptor family pyrin domain-containing 3 (NLRP3) inflammasome [14]. In addition, ROS and damage-associated molecular patterns (DAMPs) recruit immune cells such as macrophages and neutrophils, enhancing inflammatory responses and renal interstitial fibrosis [15]. Persistent ferroptosis and chronic inflammation further activate myofibroblasts, leading to extracellular matrix (ECM) deposition, tubular atrophy, and CKD progression [16]. These findings suggest that targeting ferroptosis may represent a promising therapeutic strategy for iron overload-associated kidney injury.

Quercetin (Que), a naturally occurring flavonoid abundant in fruits and vegetables, has attracted considerable attention because of its antioxidant and anti-inflammatory properties [17]. As a potent dietary antioxidant and free radical scavenger, Que has demonstrated protective effects against iron overload-induced organ injury. Emerging evidence indicates that Que can regulate ferroptosis by reducing ROS generation, inhibiting lipid peroxidation, and restoring iron homeostasis [18]. More critically, a substantial body of experimental evidence has characterized the iron-chelating properties of Que, with multiple independent studies confirming its ability to form stable coordination complexes with Fe^2+^ and Fe^3+^ via the 3′,4′-dihydroxy groups in the B ring and the 3-hydroxy-4-keto groups in the C ring [19]. This metal-chelating capacity provides an additional mechanistic basis for the protective effects of Que against iron overload-induced oxidative damage and ferroptosis. Notably, Que has shown nephroprotective effects through modulation of antioxidant defense pathways, including nuclear factor erythroid-derived 2-related factor 2 (NRF2), SLC7A11 (xCT), and GPX4 signaling, which are critical for cellular resistance to oxidative stress and ferroptosis [20,21].

In summary, the kidneys are both regulators of iron metabolism and victims of abnormal iron metabolism. Ferrostatin-1 (Fer-1) is one of the most widely used ferroptosis inhibitors; however, its poor in vivo stability limits its clinical applicability [22]. Therefore, identifying naturally derived compounds with anti-ferroptotic activity and improved safety profiles remains an important research objective. In the present study, we aimed to elucidate the molecular mechanisms underlying iron overload-induced nephrotoxicity and to investigate the protective effects of Que against ferroptosis-mediated renal injury. We hypothesized that Que attenuates iron overload-induced kidney damage by restoring redox balance, maintaining iron homeostasis, and activating the NRF2/xCT/GPX4 antioxidant signaling pathway.

## 2. Materials and Methods

### 2.1. Animal Experiment

The experiment was conducted with male C57BL/6J mice (20–22 g, 6–8 week), poured from Bena Culture Collection (Beijing, China). Mice were housed in a room with a 12 h/12 h light/dark cycle, and habituated in the room 7 days before experiments. In the study, randomly divide 15 healthy mice into three groups. Drug treatment was performed based on the literature: The first group was treated with a 0.5% Carboxymethyl Cellulose Sodium (CMC-Na) + 0.9% Nacl injection (*n* = 5), The second group was treated with a 0.5% CMC-Na + iron dextran (ID, Sigma, St. Louis, MO, USA) injection (*n* = 5), The third group was treated with a 100 mg/kg Que + ID injection (*n* = 5) [23]. The solvent for Que in vivo was 0.5% CMC-Na, respectively. Mice were continuously treated with the above-mentioned drugs for 7 days. Samples were collected via euthanasia 24 h after the final injection. All animal procedures were approved by the Animal Ethics Committee of the North Henan Medical University.

### 2.2. Serum Renal Function Index

Take a mouse blood sample and centrifugated at 3500 rpm for 15 min to obtain the serum. According to the Creatinine (CRE) and Blood Urea Nitrogen (BUN) kit instructions (Nanjing Jiancheng Bioengineering Institute, Nanjing, China), serum CRE and BUN levels were measured using standard colorimetric assays to monitor renal function.

### 2.3. Histological Analysis

Renal tissues were fixed in 4% paraformaldehyde, paraffin-embedded, and sectioned at 4 μm. After deparaffinization and rehydration, sections were stained with hematoxylin and eosin (H&E) for morphological evaluation. PAS staining was performed by incubating sections with Schiff reagent (Solarbio, Beijing, China) for 20 min followed by hematoxylin counterstaining. Slides were dehydrated, mounted with neutral balsam, and observed under a light microscope (TANON, Shanghai, China) to assess mesangial matrix expansion and renal tubular injury.

### 2.4. Terminal Deoxynucleotidyl Transferase dUTP Nick End Labeling

The impact of iron overload on renal cell viability was evaluated by TUNEL staining on 10 μm frozen kidney sections using a Solarbio kit (Beijing, China). The staining procedure included deparaffinization, antigen retrieval, and permeabilization steps. Following incubation with the TUNEL reaction mixture, the sections were rigorously washed with PBS between each step. Nuclei were counterstained with DAPI, and the slides were mounted for analysis. Apoptotic cells were finally detected and quantified under a fluorescence microscope (Leica, Wetzlar, Germany).

### 2.5. Cell Culture and Treatment

HK-2 cells were attained from the China Cell Culture Center (Shanghai, China). The cells were cultured in RPMI/1640 (Solarbio, Beijing, China) supplemented with 10% fetal bovine serum (HyClone, Logan, UT, USA) and antibiotics (HyClone) under a humidified incubator containing 5% CO_2_ at 37 °C.

Before treatment with Ferric citrate (FAC, Solarbio, Beijing, China), cells were pretreatment with 1 μM ferroptosis specific inhibitor Fer-1 (Glpbio, Montclair, CA, USA) and 10 μM deferoxamine mesylate (DFO, Glpbio, Montclair, CA, USA), or 1 μM Heme oxygenase 1 (HO1) inhibitor zinc protoporphyrin IX (ZnPP, Glpbio, Montclair, CA, USA) for 1 h [23]. For mechanistic investigations, cells were additionally treated with the ferroptosis inducers RSL3 (0.5 μM) and erastin (1 μM), the apoptosis inhibitor Z-VAD-FMK (20 μM), and the necroptosis inhibitor necrostatin-1 (Nec-1, 100 μM). All compounds were obtained from Beyotime (Shanghai, China) unless otherwise indicated.

### 2.6. Cell Counting Kit-8 Assay

HK-2 cells were inoculated on 96-well plates. Following a 24 h treatment period, the cells were subjected to experimental conditions as detailed in the corresponding figure legends. Cell viability was subsequently determined using the Cell Counting Kit-8 (CCK8, Solarbio, Beijing, China) following the manufacturer’s recommended protocol.

### 2.7. Measurement of Mitochondrial Membrane Potential

HK-2 cells were inoculated in a 6-well plate, the mitochondrial membrane potential of HK-2 cells was labeled with 200 nM Mito-Tracker Red CMXRos (Beyotime, Shanghai, China), and incubated in an incubator at 37 °C for 20 min. The staining results were observed using a fluorescence microscope according to the manufacturer’s instructions.

### 2.8. Mitochondrial Superoxide Assay

HK-2 cells were incubated with 10 μM MitoNeoD at 37 °C in a humidified incubator containing 5% CO_2_ for 30 min. After staining, cells were washed three times with PBS to remove excess probe. Mitochondrial superoxide production was detected using an fluorescence microscope.

### 2.9. Calcein-AM/Prodipium Iodine (PI) Staining

HK-2 cells were inoculated in a 6-well plate, and the effects of drugs on the viability of HK-2 cells were detected by Calcein-AM/PI Live/Dead Viability Assay Kit (Solarbio, Beijing, China). According to the manufacturer’s instructions, HK-2 cells were incubated in a containing 1 mL of 1 × Assay Buffer containing 2 μM Calcein-AM and 5 μM PI for 30 min. The staining results were observed by fluorescent microscope.

### 2.10. Perls’ Blue Staining

Frozen kidney sections and cell crawling slices with a thickness of 10 μm were subjected to iron staining using a Prussian Blue Iron Stain Kit (Solarbio, Beijing, China) to visualize the iron content and distribution within the kidney tissue and cells. All experimental procedures were strictly performed in accordance with the manufacturer’s protocol.

### 2.11. Measurement of Iron Content

Use commercially available assay kits (Nanjing Jiancheng Bioengineering Institute, Nanjing, China) to quantify serum iron concentration, as well as iron levels in tissues and cells, in strict accordance with the manufacturer’s protocols.

### 2.12. Measurement of Glutathione (GSH) and Malondialdehyde (MDA) Content

The concentrations of GSH and MDA in renal tissues and HK-2 cell lysates were quantitatively determined using colorimetric assay kits (Nanjing Jiancheng Bioengineering Institute, Nanjing, China) in strict accordance with the manufacturer’s standardized protocols.

### 2.13. Assessment of ROS

The average level of superoxide anion was detected by dihydroethidium (DHE, Beyotime, Shanghai, China) in kidney. The Frozen sections (10 μm) were washed twice in PBS and then loaded with DHE (5 μmol/L) in incubator at 37 °C in the dark for 30 min. Images were obtained with the fluorescence microscope.

The Dichlorodihydrofiuorescein diacetate (DCFH-DA) probe (Beyotime, Shanghai, China) was used to quantify ROS levels in HK-2 cells. The HK-2 cells were seeded into the 6-well plates and maintained under standard culture conditions for 24 h. In accordance with the manufacturer’s protocol, the cells were rinsed with PBS, subsequently treated with 10 μM DCFH-DA, incubated at 37 °C in a humidified atmosphere containing 5% CO_2_ for 30 min, and ultimately visualized using fluorescence microscopy.

### 2.14. Lipid Peroxidation Assay

After drug treatment, cells were incubated with 2 μM BODIPY™ 581/591 C11 lipid peroxidation sensor (Beyotime, Shanghai, China) at 37 °C in a humidified incubator with 5% CO_2_ for 30 min in the dark. Cells were then washed three times with PBS to remove excess probe. Lipid peroxidation was evaluated by fluorescence microscopy according to the manufacturer’s instructions.

### 2.15. Real-Time Quantitative Polymerase Chain Reaction

Total RNA was extracted from HK-2 cells and kidney tissue using TRIzol reagent (Thermo Fisher Scientific, Waltham, MA, USA), followed by reverse transcription into cDNA using a Reverse Transcription Kit (Takara, Tokyo, Japan) according to the manufacturer’s protocol. Quantitative real-time PCR (RT-qPCR) analysis was performed using a qPCR instrument. qPCR was executed as in our previous report [24]. The relative gene expression levels were normalized to β-actin as an internal reference and calculated using the 2^−ΔΔCt^ method. All primer sequences used in this study are provided in the Supplementary Materials.

### 2.16. Western Blotting

Western blotting was performed as described [25], the following primary antibodies were used for analysis: SLC7A11/xCT (Cell Signaling Technology, Danvers, MA, USA; cat. no. 98051), Nuclear factor erythroid-derived 2 (NRF2, CST, cat. no. 12721), Ferritin (FTH, CST, cat. no. 4393), GPX4 (CST, cat. no. 52455), Transferrin (TF, Proteintech Group, cat. no. 17435-1-AP), HO1 (Proteintech, cat. no. 10701-1-AP), Transferrin receptor1 (TFR, Abcam, Cambridge, MA, USA; cat. no. ab214039), and β-actin (Proteintech, cat. no. 66009-1-Ig). HRP conjugated secondary goat anti-rabbit and goat anti-mouse antibodies (Beyotime, cat. no. A0216 and A0208) were diluted according to the manufacturer’s instructions. Finally, the images of Western blotting are analyzed using Bio-Rad’s Image Lab software. The densitometry of protein bands was quantified using Image J software (Image J v1.46a, National Institutes of Health, Bethesda, MD, USA).

### 2.17. Statistical Analysis

All experimental data are presented as mean ± standard error of the mean (SEM). Statistical analyses were performed using either unpaired two-tailed Student’s *t*-test or one-way analysis of variance (ANOVA), as appropriate. A probability value of *p* < 0.05 was considered statistically significant, with specific significance levels denoted as follows: * *p* < 0.05. All cell-based experiments were conducted in triplicate to ensure reproducibility. Data analysis and graphical representations were generated using GraphPad Prism 8.0 software (GraphPad Software, San Diego, CA, USA).

## 3. Results

### 3.1. Iron Overload Contributes to Renal Dysfunction

**Figure 1:**
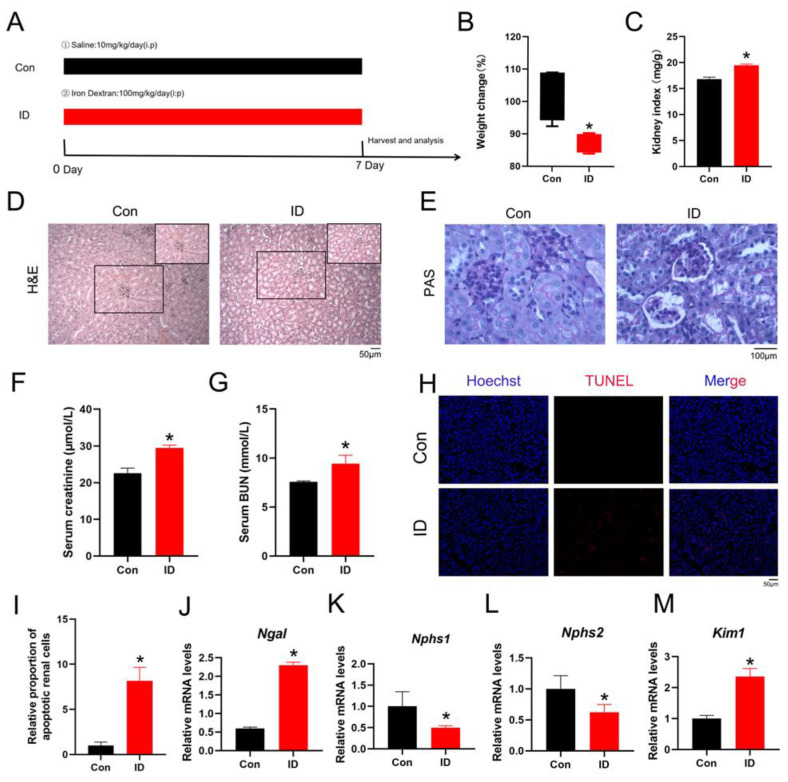
The effect of iron overload on the structure and function of mouse kidneys. (**A**) Animal experiment design. Mice were intraperitoneally (i.p.) administered ID at 100 mg/kg per day, for 7 days, while control mice were given saline i.p (*n* = 5). (**B**) Changes in the body weight of mice in each group during the experiment (*n* = 5). (**C**) Changes in kidney organ index of each group of mice (*n* = 5). (**D**,**E**) H&E and PAS staining results in kidney tissue. (**F**,**G**) The content of serum CRE and BUN (*n* = 5). (**H**,**I**) TUNEL staining and quantitative analysis of kidney tissue sections (*n* = 3). (**I**–**M**) Analysis of mRNA levels of relative genes by qPCR (*n* = 3). Data are presented as mean ± SEM. * *p* < 0.05 vs. the control group.

To evaluate the toxic effects of iron overload on renal function, a mouse model of iron overload-induced kidney injury was established (Figure 1A). One week after treatment, mice in the ID group exhibited a significant reduction in body weight compared with controls (Figure 1B). Consistently, the kidney index (kidney weight/body weight ratio) was significantly increased in ID-treated mice (Figure 1C). Histopathological examination further confirmed renal injury. H&E and PAS staining revealed glomerular enlargement, tubular cystic dilation, and tubular epithelial cell desquamation in the kidneys of ID-treated mice (Figure 1D,E). In parallel, serum CRE and BUN levels were markedly elevated, indicating impaired renal function (Figure 1F,G). TUNEL staining demonstrated a significant increase in renal cell death following iron overload (Figure 1H,I). Consistent with these findings, quantitative PCR analysis showed that the acute kidney injury markers Neutrophil Gelatinase-Associated Lipocalin (*Ngal*) and *Kim1* (*Havcr1*) were significantly upregulated in the ID group, indicating renal tubular injury. In addition, the expression of podocyte-associated genes was markedly reduced. Specifically, Nephrin (*Npsh1*) and Podocin (*Npsh2*) were significantly downregulated after ID treatment, suggesting structural and functional impairment of glomerular podocytes (Figure 1J–M). Collectively, these results demonstrate that iron overload induces renal dysfunction characterized by tubular injury, podocyte damage, and increased renal cell death.

### 3.2. Iron Overload Induces Iron Metabolic Imbalance and Ferroptosis-Related Changes in the Kidney

**Figure 2:**
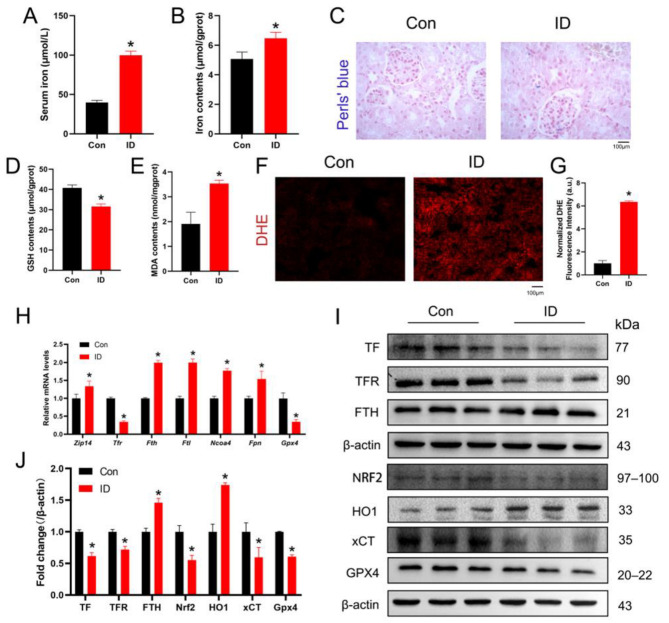
ID induced ferroptosis in mouse kidney. (**A**,**B**) The levels of serum iron and kidney iron (*n* = 3). (**C**) Perls’ Prussian blue staining for iron in kidney. (**D**,**E**) Kidney GSH and MDA content in control and ID injected mice (*n* = 5). (**F**,**G**) Representative images and quantitative analysis of Dihydroethidium staining in kidney tissue sections (*n* = 3). (**H**) Analysis of mRNA levels of relative genes by qPCR (*n* = 3). (**I**,**J**) Western blotting analysis of ferroptotic proteins in mice and semi-quantitative analysis of Western blotting (*n* = 3). Data are presented as mean ± SEM. * *p* < 0.05 vs. the control group.

To investigate the effects of iron overload on renal iron metabolism and ferroptosis-related processes, we examined iron accumulation, oxidative stress markers, and ferroptosis-associated gene and protein expression in kidney tissues. As expected, iron overload markedly increased iron levels in both serum and renal tissues (Figure 2A,B). Consistently, Perls’ Prussian blue staining revealed prominent iron deposition in renal tissues of ID-treated mice (Figure 2C). Iron overload also induced pronounced oxidative stress and lipid peroxidation. Compared with controls, GSH levels were significantly decreased, whereas MDA levels were increased in kidney tissues (Figure 2D,E), indicating disruption of redox homeostasis. These findings were further supported by enhanced ROS production detected by DHE staining (Figure 2F,G). To further evaluate alterations in iron metabolism and ferroptosis-related pathways, gene expression was analyzed by qPCR. The results showed significant upregulation of Solute carrier family 39 member A14 (*SLC39A14* or *Zip14*), *Fth*, *Ftl*, Nuclear Receptor Coactivator 4 (*Ncoa4*), and Ferroportin (*Fpn*), whereas *Tfr* and *Gpx4* were significantly downregulated (Figure 2H), suggesting dysregulated iron handling and impaired antioxidant defense. Consistent with the transcriptional changes, Western blot analysis demonstrated increased protein levels of FTH and HO1, while TF, TFR, NRF2, xCT, and GPX4 were markedly decreased in the kidneys of ID-treated mice (Figure 2I,J). Collectively, these findings indicate that iron overload disrupts renal iron homeostasis, enhances oxidative stress and lipid peroxidation, and promotes ferroptosis-associated molecular alterations in the kidney.

### 3.3. Iron Overload Induces Ferroptosis in Renal Tubular Cells

**Figure 3:**
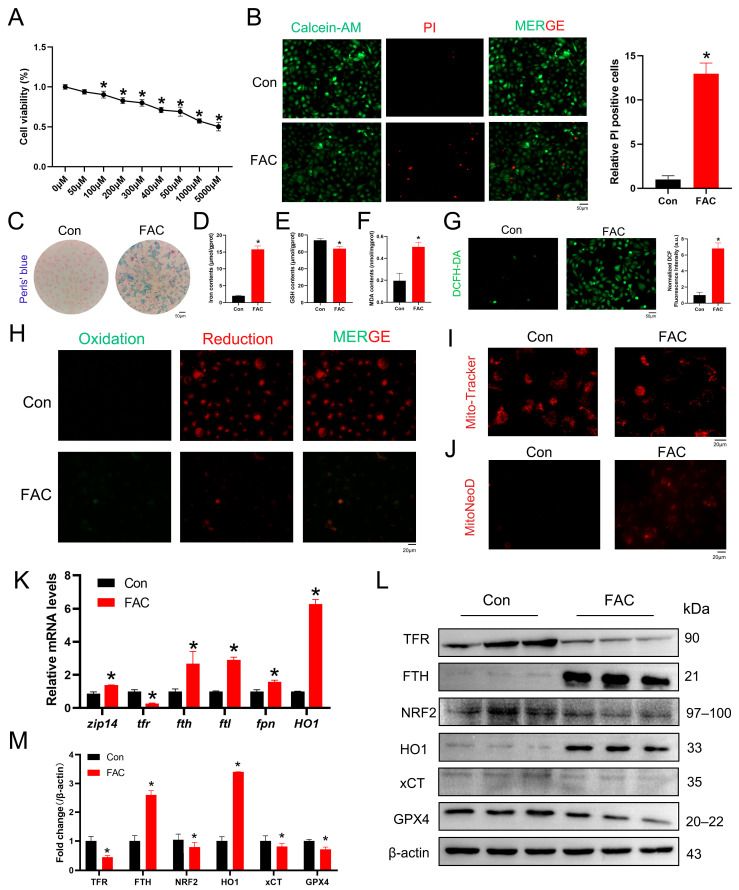
Iron overload-induced ferroptosis in HK-2 cells. (**A**) HK-2 cells were treated with different concentrations of FAC for 24 h, and cell viability was assayed using CCK-8 (*n* = 5). (**B**) Calcein-AM/PI staining of HK-2 cells exposed to FAC and the proportion of PI-positive cells (5 mM). (**C**) Perls’ Prussian blue staining for iron in HK-2 cells. (**D**) Iron content after FAC exposure in HK-2 cells (*n* = 5). (**E**,**F**) Changes in GSH and lipid peroxidation (MDA) levels in HK-2 cells after exposure to FAC (*n* = 5). (**G**) The cellular reactive oxygen species (ROS) levels in HK-2 cells exposed to FAC for 24 h were assessed using the DCFH-DA probe, along with quantitative analysis (*n* = 3). (**H**) Representative images of lipid ROS level in HK-2 cells with BODIPY 581/591 C11 (*n* = 3). (**I**) Representative microscopic images of HK-2 cells stained with Mito-Tracker Red (*n* = 3). (**J**) Mitochondrial superoxide production was detected by the MitoNeoD probe. (*n* = 3). (**K**) qPCR of ferroptotic genes in HK-2 cells after FAC exposure (*n* = 3). (**L**,**M**). Western blotting of ferroptotic proteins after FAC exposure in HK-2 cells (*n* = 3). β-actin was used as the reference protein. Data are presented as mean ± SEM. * *p* < 0.05 vs. the control group.

To establish an in vitro model of iron overload-induced ferroptosis, HK-2 cells were treated with FAC. Cell viability was first evaluated using a CCK-8 assay across a concentration gradient of FAC. Based on the dose–response curve, 200 μM FAC was selected as the optimal concentration for subsequent experiments (Figure 3A). Calcein-AM/PI staining further demonstrated a marked increase in cell death following FAC treatment (Figure 3B), confirming successful induction of ferroptosis-related injury. Because iron accumulation and lipid peroxidation are central features of ferroptosis, we next assessed intracellular iron and oxidative stress. Perls’ staining revealed increased iron deposition in FAC-treated HK-2 cells (Figure 3C). Consistently, FAC exposure resulted in intracellular iron accumulation, GSH depletion, and elevated MDA levels (Figure 3D–F). ROS production detected by DCFH-DA fluorescence was significantly increased following FAC treatment (Figure 3G). Using the lipid peroxidation probe BODIPY 581/591 C11, we observed a pronounced elevation in lipid ROS levels in HK-2 cells (Figure 3H). Mitochondrial dysfunction was also evident. Mito-Tracker Red staining indicated collapse of mitochondrial membrane potential after FAC exposure (Figure 3I), accompanied by increased mitochondrial superoxide production detected using MitoNeoD (Figure 3J). To further characterize ferroptosis-related molecular changes, qPCR analysis showed upregulation of *ZIP14*, *FTH*, *FTL*, *FPN*, and *HO1*, whereas *TFR* expression was significantly reduced following FAC treatment (Figure 3K). Western blot analysis demonstrated decreased protein levels of TFR, NRF2, xCT, and GPX4, together with increased FTH and HO1 expression (Figure 3L,M). Collectively, these results indicate that FAC induces ferroptosis-associated oxidative stress, mitochondrial dysfunction, and dysregulation of iron metabolism in renal tubular cells.

To verify that ferroptosis mediates FAC-induced cytotoxicity, cells were treated with the ferroptosis inhibitor Fer-1 and the iron chelator deferoxamine (DFO). Both Fer-1 (1 μM) and DFO (10 μM) significantly restored cell viability (Figure S1A). In contrast, the apoptosis inhibitor Z-VAD-FMK and the necrosis inhibitor Necrostatin-1 failed to rescue FAC-induced cell death (Figure S2), further supporting that ferroptosis is the primary mode of cell death under iron overload conditions. FAC-induced ROS accumulation detected by DCFH-DA staining was markedly attenuated by both inhibitors (Figure S1B,E). Similarly, mitochondrial membrane potential was restored (Figure S1C), and Calcein-AM/PI staining confirmed reduced cell death (Figure S1D,F). Western blot analysis further showed that Fer-1 reversed FAC-induced FTH and HO1 upregulation and restored TFR, NRF2, xCT, and GPX4 expression (Figure S1G).

Given the emerging role of HO1 in ferroptosis regulation, we next examined its involvement in this model. Western blot analysis demonstrated that FAC treatment induced a dose-dependent increase in HO1 protein expression (Figure S3A). Pharmacological inhibition of HO1 using ZnPP significantly improved cell viability following FAC exposure (Figure S3B). ZnPP treatment also reduced intracellular iron accumulation (Figure S3C), decreased ROS production (Figure S3D,G), restored mitochondrial membrane potential, and attenuated FAC-induced cell death (Figure S3E,F,H). Furthermore, ZnPP reversed the FAC-induced downregulation of TFR, NRF2, xCT, and GPX4, as well as the upregulation of FTH and HO1 (Figure S3G). Together, these results demonstrate that iron overload induces ferroptosis in HK-2 cells and that HO1 signaling contributes to this process.

### 3.4. Que Protects Kidney the from Iron Overload-Induced Renal Dysfunction

**Figure 4:**
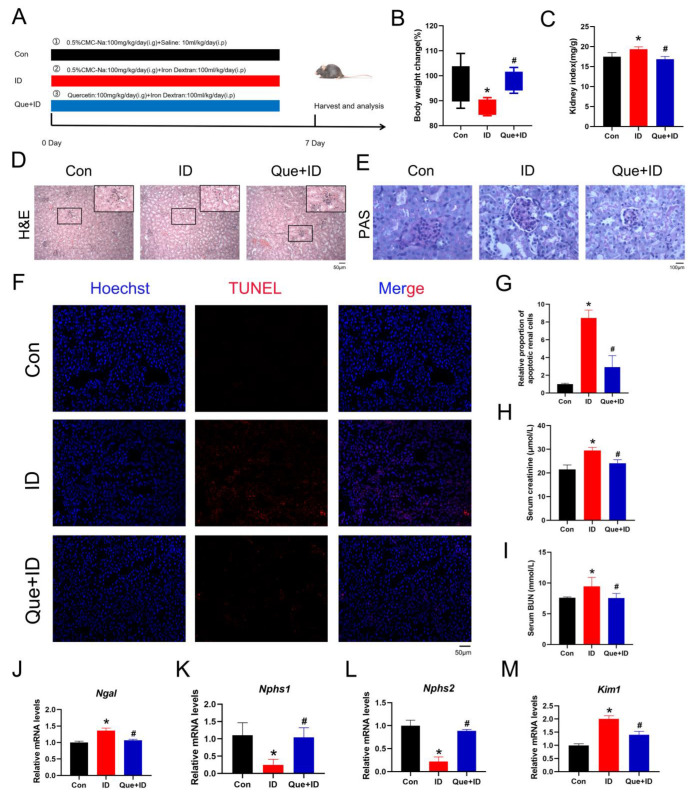
Que improves ID-induced renal dysfunction. (**A**) Animal experiment design (*n* = 5). (**B**) Changes in the body weight of mice in each group during the experiment (*n* = 5). (**C**) Changes in kidney organ index of each group of mice (*n* = 5). (**D**,**E**) H&E and PAS staining results in kidney tissue. (**F**,**G**) TUNEL staining and quantitative analysis of kidney tissue sections (*n* = 3). (**H**,**I**) The content of serum CRE and BUN (*n* = 5). (**J**–**M**) Analysis of mRNA levels of relative genes by qPCR (*n* = 3). Data are presented as mean ± SEM. * *p* < 0.05 vs. the control group. # *p* < 0.05 vs. the ID group.

To evaluate the protective effects of Que against iron overload–induced renal injury, mice were treated with Que following ID administration (Figure 4A). As shown in Figure 4B, Que treatment partially restored body weight loss induced by iron overload. Consistently, the renal index was significantly reduced in the Que-treated group compared with the ID group, approaching normal levels (Figure 4C). Histopathological examination further supported the protective role of Que. H&E and PAS staining demonstrated that Que markedly alleviated iron overload–induced renal structural damage, including glomerular enlargement, tubular dilation, basement membrane irregularity, and tubular epithelial cell detachment (Figure 4D,E). TUNEL staining revealed that Que significantly reduced renal cell death induced by iron overload (Figure 4F,G). Biochemical analyses showed that Que treatment significantly decreased serum CRE and BUN levels compared with the ID group (Figure 4H,I), indicating improved renal function. Consistently, qPCR analysis demonstrated that Que reversed ID-induced alterations in renal injury–related gene expression. Specifically, Que reduced the elevated expression of *Ngal* and *Kim1*, while restoring the expression of podocyte-associated genes *Nphs1* and *Nphs2* (Figure 4J–M). Collectively, these findings indicate that Que effectively attenuates iron overload–induced renal dysfunction and tissue injury in vivo.

### 3.5. Que Alleviates Renal Injury by Inhibiting Ferroptosis

**Figure 5:**
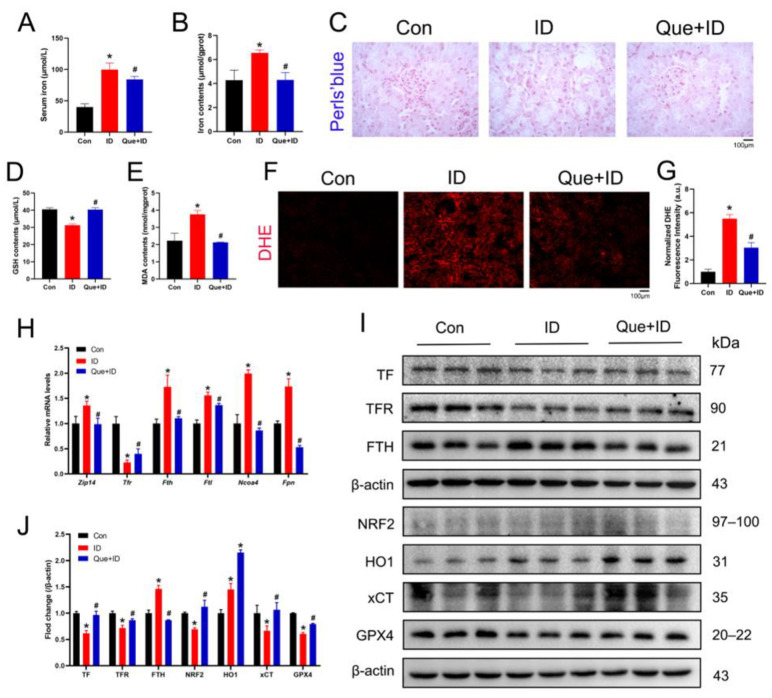
Que alleviates kidney injury by inhibiting ferroptosis. (**A**,**B**) The levels of serum iron and kidney iron (*n* = 3). (**C**) Perls’ Prussian blue staining for iron in kidney. (**D**,**E**) Kidney GSH and MDA content after Que treatment (*n* = 5). (**F**,**G**) Representative images and quantitative analysis of Dihydroethidium staining in kidney tissue sections (*n* = 3). (**H**) Analysis of mRNA levels of relative genes by qPCR (*n* = 3). (**I**,**J**) Western blotting analysis of ferroptotic proteins in mice and semi-quantitative analysis of Western blotting (*n* = 3). Data are presented as mean ± SEM. * *p* < 0.05 vs. the control group. # *p* < 0.05 vs. the ID group.

To determine whether the protective effect of Que against iron overload–induced renal injury is associated with suppression of ferroptosis, key ferroptosis-related indicators were evaluated in renal tissues. Compared with the control group, ID-treated mice exhibited markedly increased iron levels in both serum and kidney tissues. Que administration significantly reduced iron accumulation relative to the ID group (Figure 5A,B). Consistently, Perls’ blue staining demonstrated a clear reduction in iron deposition in renal tissues following Que treatment (Figure 5C). Oxidative stress markers were further examined. Que treatment restored GSH levels and reduced MDA accumulation in kidney tissues compared with ID-treated mice (Figure 5D,E). DHE staining revealed that Que markedly suppressed iron overload–induced ROS production in renal tissues (Figure 5F,G). To further assess the regulatory effects of Que on iron metabolism and ferroptosis-related pathways, gene expression analysis was performed. qPCR results showed that Que attenuated the ID-induced upregulation of *Zip14*, *Fth*, *Ftl*, *Ncoa4*, and *Fpn*, while restoring *Tfr* expression (Figure 5H). At the protein level, Western blot analysis demonstrated that Que treatment increased the expression of ferroptosis-protective proteins, including TF, TFR, NRF2, xCT, and GPX4, while reducing FTH expression compared with the ID group (Figure 5I,J). Regulation of these ferroptosis-associated proteins suggests restoration of iron homeostasis and antioxidant defense. Collectively, these findings indicate that Que mitigates iron overload–induced renal injury by suppressing ferroptosis and restoring redox balance and iron metabolism.

### 3.6. Iron Overload-Induced Ferroptosis Was Alleviated Using Que in Renal Tubular Cells

**Figure 6:**
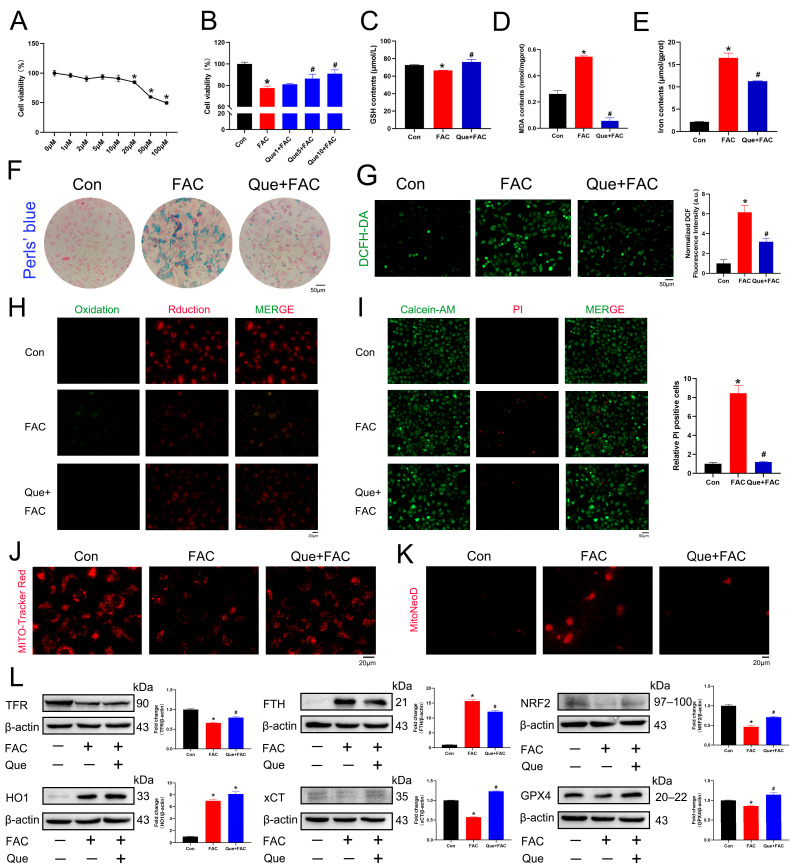
Que alleviates ferroptosis in HK-2 cells. (**A**,**B**) Cell viability in HK-2 cells (*n* = 5). (**C**,**D**) The levels of GSH and MDA after Que treatment (10 μM) in HK-2 cells (*n* = 5). (**E**) Iron content after Que treatment in HK-2 cells (*n* = 5). (**F**) Perls’ Prussian blue staining for iron in HK-2 cells. (**G**) Assessment of cellular ROS levels and quantitative analysis in HK-2 cells using the DCFH-DA probe (*n* = 3). (**H**) Representative images of lipid ROS level in HK-2 cells with BODIPY 581/591 C11 (*n* = 3). (**I**) Calcein-AM/PI staining of HK-2 cells following Q treatment and the proportion of PI-positive cells (*n* = 3). (**J**) Representative microscopic images of HK-2 cells stained with Mito-Tracker Red (*n* = 3). (**K**) Mitochondrial superoxide production was detected by the MitoNeoD probe. (*n* = 3). (**L**). Western blotting of ferroptotic proteins after Q treatment in HK-2 cells. β-actin was used as the reference protein (*n* = 3). Data are presented as mean ± SEM. * *p* < 0.05 vs. the control group. # *p* < 0.05 vs. the FAC group.

**Figure 7:**
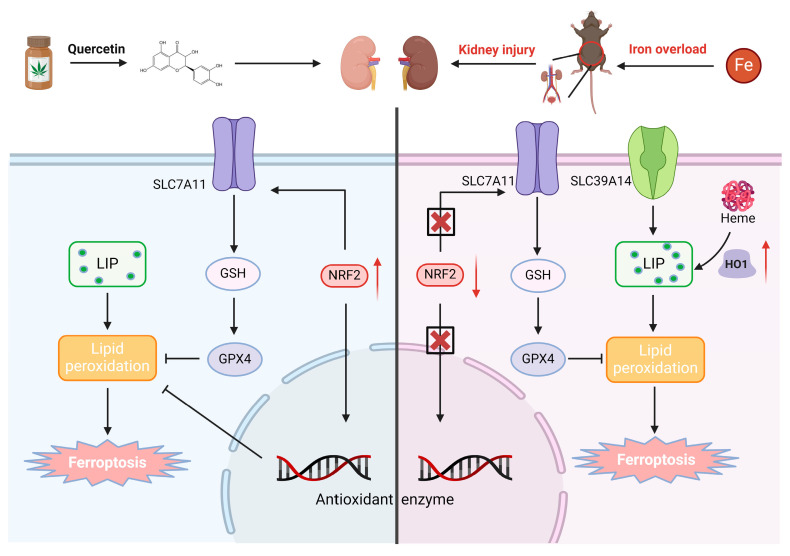
Schematic diagram for Que attenuated renal injury induced by iron overload (↑, Increase; ↓, Decrease).

To determine a suitable non-toxic concentration of Que for in vitro experiments, HK-2 cells were treated with increasing concentrations of Que (0–100 μM), and cell viability was evaluated using a CCK-8 assay. Que concentrations up to 10 μM showed no significant cytotoxicity. Therefore, 10 μM Que was selected for subsequent experiments (Figure 6A). Next, the protective effect of Que on FAC-induced cellular injury was assessed. CCK-8 analysis demonstrated that Que treatment (0–10 μM) dose-dependently improved HK-2 cell viability following FAC exposure (Figure 6B). To evaluate ferroptosis-related changes, oxidative stress and lipid peroxidation markers were examined. Que treatment restored intracellular GSH levels and reduced MDA accumulation in FAC-treated HK-2 cells (Figure 6C,D). Consistently, intracellular iron accumulation induced by FAC was significantly reduced following Que treatment (Figure 6E,F). DCFH-DA staining showed that Que markedly attenuated FAC-induced ROS generation (Figure 6G), and BODIPY 581/591 C11 staining confirmed suppression of lipid ROS accumulation (Figure 6H). Cell death analysis further supported these findings. Calcein-AM/PI staining demonstrated that Que significantly rescued FAC-induced HK-2 cell death (Figure 6I). In addition, Que restored mitochondrial membrane potential disrupted by FAC treatment (Figure 6J) and reduced mitochondrial superoxide production (Figure 6K). To further investigate the molecular mechanisms, ferroptosis-related protein expression was analyzed by Western blot. FAC treatment decreased the expression of TFR, NRF2, xCT, and GPX4, while increasing FTH and HO1 expression. Que treatment largely reversed these alterations, restoring TFR, NRF2, xCT, and GPX4 levels while reducing FTH expression. Notably, HO1 expression remained elevated after Que treatment (Figure 6L). To further verify the anti-ferroptotic effect of Que, HK-2 cells were exposed to ferroptosis inducers RSL3 and erastin. As shown in Figure S4, Que significantly rescued cell viability under both conditions, further supporting its role as a ferroptosis-modulating agent. Collectively, these results demonstrate that Que alleviates iron overload–induced ferroptosis in renal tubular epithelial cells by restoring redox balance, mitochondrial function, and ferroptosis-related signaling pathways (Figure 7).

## 4. Discussion

Ferroptosis research has expanded rapidly since its initial description as an iron-dependent form of regulated cell death characterized by lipid peroxidation. Increasing evidence indicates that ferroptosis plays a critical role in the pathogenesis of renal diseases, including acute kidney injury (AKI) and CKD [26,27]. The kidney is particularly susceptible to ferroptotic damage because of its high metabolic activity, abundant mitochondrial content, and sensitivity to oxidative stress. Iron overload is recognized as a major trigger of ferroptosis, primarily through the accumulation of redox-active Fe^2+^, which promotes lipid peroxidation and oxidative injury [28]. Understanding how iron overload induces renal ferroptosis is therefore essential for developing effective therapeutic strategies.

In the present study, we established both in vivo and in vitro models of iron overload-induced renal injury and demonstrated that ferroptosis is a key mechanism underlying nephrotoxicity. Mice exposed to ID for seven days exhibited reduced body weight, decreased vitality, and impaired renal function, as reflected by elevated serum CRE and BUN levels. Iron overload also resulted in abnormal iron accumulation and increased lipid peroxidation in renal tissues. Consistently, FAC treatment reduced HK-2 cell viability in vitro. Importantly, ferroptosis inhibitors, including Fer-1 and DFO, attenuated lipid peroxidation and mitochondrial dysfunction, supporting the conclusion that ferroptosis mediates iron overload-induced renal injury.

Iron metabolism is tightly regulated to maintain systemic iron homeostasis, and disruption of this balance is closely linked to ferroptosis susceptibility [29,30]. The accumulation of Fe^2+^ and the resulting lipid peroxidation cascade are central features of ferroptosis [31]. In our study, both in vivo and in vitro experiments demonstrated abnormal iron accumulation under iron overload conditions. This was accompanied by oxidative stress and activation of ferroptosis-related molecular pathways. Specifically, xCT and GPX4 protein levels were significantly decreased, together with depletion of GSH and elevation of MDA levels in renal tissues and FAC-treated HK-2 cells. Meanwhile, NRF2 expression was markedly reduced, indicating impaired antioxidant defense capacity. These findings suggest that iron overload promotes ferroptosis by disrupting the NRF2/xCT/GPX4 antioxidant axis and redox homeostasis.

Changes in iron metabolism-related genes further supported this conclusion. In iron-overloaded mouse kidneys, qPCR analysis revealed increased expression of *Zip14*, *Fth*, *Ncoa4*, and *Fpn*. Similarly, FAC-treated HK-2 cells showed elevated expression of *ZIP14*, *FTH*, and *FPN*. These transcriptional changes likely represent compensatory responses to intracellular iron imbalance but may also contribute to ferroptosis progression by altering iron transport, storage, and recycling processes. Interestingly, *TFR* expression was significantly reduced in iron-overloaded kidneys and FAC-treated HK-2 cells. This may represent a negative feedback response to intracellular iron accumulation. Under iron-replete conditions, suppression of IRP activity reduces *TFR* mRNA stability, thereby limiting further iron uptake. Such compensatory downregulation of *TFR* has been reported in iron overload models and reflects disruption of iron homeostasis [32]. Despite this adaptive response, intracellular iron remained elevated, indicating that iron overload exceeded the buffering capacity of cellular regulatory systems and contributed to ferroptosis progression.

HO1 represents another important regulator of intracellular iron homeostasis. HO1 degrades heme into biliverdin, carbon monoxide, and Fe^2+^, thereby influencing iron recycling and storage [33]. Previous studies have demonstrated that HO1 exerts context-dependent effects in ferroptosis. On one hand, HO1 activation can enhance antioxidant defense; on the other hand, excessive HO1 activity may increase intracellular free iron levels and promote lipid peroxidation [34,35]. HO1 can also regulate the expression of iron metabolism-related proteins, including iron transporters, thereby influencing cellular iron flux [36,37]. In this study, HO1 expression was significantly increased in both iron-overloaded kidneys and FAC-treated HK-2 cells, whereas NRF2 and GPX4 were downregulated. Pharmacological inhibition of HO1 using ZnPP effectively attenuated ferroptosis, suggesting that HO1 contributes to ferroptosis progression under iron overload conditions. These findings indicate that excessive HO1 activation may exacerbate iron accumulation and oxidative stress, thereby promoting renal ferroptosis.

Que, a widely distributed dietary flavonoid, has attracted considerable attention because of its antioxidant and anti-inflammatory properties [38]. Previous studies have shown that Que can regulate ferroptosis through multiple mechanisms, including free radical scavenging, modulation of antioxidant signaling pathways, and regulation of iron metabolism [39]. Structurally, the polyphenolic backbone of Que enables direct scavenging of lipid radicals and neutralization of toxic lipid peroxidation products, thereby interrupting the ferroptotic cascade. Consistent with these reports, our study demonstrated that Que significantly alleviated iron overload-induced renal injury in vivo. Que treatment reduced CRE and BUN levels, improved body weight loss, and mitigated histological damage in renal tissues. At the molecular level, Que reduced intracellular iron accumulation, decreased MDA levels, restored GSH content, and suppressed ROS production. These findings indicate that Que effectively inhibits ferroptosis in iron-overloaded kidneys. Mechanistically, Que restored antioxidant defense by activating the NRF2/xCT/GPX4 signaling pathway. NRF2 is a master regulator of cellular redox homeostasis and plays a crucial role in ferroptosis resistance [40]. Activation of NRF2 promotes expression of xCT, enhances cystine uptake and GSH synthesis, and sustains GPX4 activity, thereby preventing lipid peroxidation. In our in vivo and in vitro experiments, Que markedly increased NRF2, xCT, and GPX4 expression, supporting its role in restoring antioxidant capacity.

Que also influenced iron metabolism-related proteins. Treatment with Que modulated the expression of TF, TFR, and ferritin, suggesting improved intracellular iron handling. In addition, Que reduced ROS generation and attenuated FAC-induced HK-2 cell injury, further confirming its anti-ferroptotic activity. The in vitro experiments corroborated the in vivo findings. FAC-induced iron overload in HK-2 cells resulted in increased lipid peroxidation, ROS accumulation, and ferroptotic cell death, all of which were significantly attenuated by Que treatment. These results suggest that Que protects renal cells from iron-dependent oxidative injury primarily through activation of NRF2-mediated antioxidant signaling and maintenance of iron homeostasis. Importantly, Fer-1, although widely used as a ferroptosis inhibitor, has limited clinical applicability because of poor in vivo stability. Natural compounds with anti-ferroptotic properties may therefore represent promising therapeutic alternatives. Our findings support Que as a potential candidate for mitigating ferroptosis-associated renal injury.

However, certain limitations of this study should be acknowledged. While we have demonstrated Que’s protective effects in animal models, further studies are needed to explore its long-term efficacy and safety in clinical settings. Additionally, the precise molecular mechanisms through which Que modulates ferroptosis-related pathways in different renal cell types remain to be fully elucidated. Que is known to possess metal-chelating activity and may directly influence cellular iron availability. Therefore, the protective effects observed in this study may involve both ferroptosis suppression and modulation of iron metabolism. Future studies using iron-binding assays or iron-independent ferroptosis models will help further distinguish these mechanisms.

## 5. Conclusions

In summary, this study demonstrates that Que effectively mitigates iron overload-induced renal dysfunction by attenuating ferroptosis in both in vivo and in vitro models. Our findings highlight that Que exerts protective effects through the modulation of key ferroptosis-related pathways, including NRF2, xCT, GPX4, and HO1, and through its ability to reduce iron accumulation, lipid peroxidation, and oxidative stress. These results suggest that Que may serve as a promising therapeutic agent for renal diseases associated with iron overload.

## Data Availability

The data that support the findings of this study are available from the corresponding author upon reasonable request.

## Supplementary Materials

The following supporting information can be downloaded at: https://www.mdpi.com/article/10.3390/life16030372/s1, Figure S1: FAC-induced ferroptosis was reversed using ferrostatin-1in HK-2 cells; Figure S2: Effects of apoptosis and necroptosis inhibitors on FAC-induced HK-2 cell death; Figure S3: FAC induced ferroptosis via HO1 in HK-2 cells; Figure S4: Que alleviates RSL3- and Erastin-induced ferroptotic cell death in HK-2 cells; Table S1: Primer sequences.
