# Supplementary material for: Quercetin Attenuates Iron Overload-Induced Renal Injury via Activating Nrf2/xCT/GPX4 Signaling to Inhibit Ferroptosis
Source: Life (Basel). 2026 Feb 25;16(3):372. doi: 10.3390/life16030372 (PMC13028383; doi:10.3390/life16030372)
Supplement: Supplementary file 1 [file life-16-00372-s001.zip › life-4033160-supplementary.pdf]

Supplementary Table S1. Primer sequences

Gene name	Forward primer (5’-3’)	Reverse primer (5’-3’)
m-*Slc39a14*	TTTCCCAGCCCAAGGAAG	CAAAGAGGTCTCCAGAGCTAAA
m-*Gpx4*	CCTCCCCAGTACTGCAACAG	GGCTGAGAATTCGTGCATGG
m-*Tfr*	TCGTACAGCAGCGGAAGT	TCTCCACGAGCGGAATACAG
m-*Fth*	GCCGAGAAACTGATGAAGCTGC	GCACACTCCATTGCATTCAGCC
m-*Ftl*	ACTTAGAGCAGCGCCTTGGA	AGGAGCTAACCGCGAAGAGA
m-*Fpn*	TTGGCTTTCCAACTTCAGCTAC	CAGCCTTATGCCGAAAGACC
m-*Ncoa4*	GGTGTGGCTCAATGAACAGGTC	TGGATGCTGACTTCTGCTCTGG
m-*Ngal*	CCACCACGGACTACAACCAGTT	GACTTGGCAAAGCGGGTGAAAC
m-*Nphs1*	CCACGGTTAGCACAGCAGAAGT	GGCTTGGCGATATGACACCTCT
m-*Nphs2*	CTGAGGATGGCGGCTGAGATTC	TGATGCTCCCTTGTGCTCTGTT
m-*Kim1*	ACATATCGTGGAATCACAACGAC	ACAAGCAGAAGATGGGCATTG
m-*β-actin*	TATGCTCTCCCTCACGCCATCC	GGAACCGCTCGTTGCCAATAGT
h-*HO1*	AGCTCCACCATGACAGGAACCT	TGGCAATAGAGCGAGTCAGAACC
h-*SLC39A14*	GGACGAGAAGGTCATTGTGG	GTGATCATCCAGGCCAGAGT
h-*TFR*	ATCGGTTGGTGCCACTGAATGG	ACAACAGTGGGCTGGCAGAAAC
h-*FTH*	TTCAACAGTGCTTGGACGGAA	GGTTGATCTGGCGGTTGATG
h-*FTL*	CCAGCACCGTTTTTGTGGTT	CAATTCGCGGAAGAAGTGGC
h-*FPN*	GAGACAAGTCCTGAATCTGTGCC	TTCTTGCAGCAACTGTGTCACAG
h-*GAPDH*	TGGTATCGTGGAAGGACTC	AGTAGAGGCAGGGATGATG

Supplemental figure legends

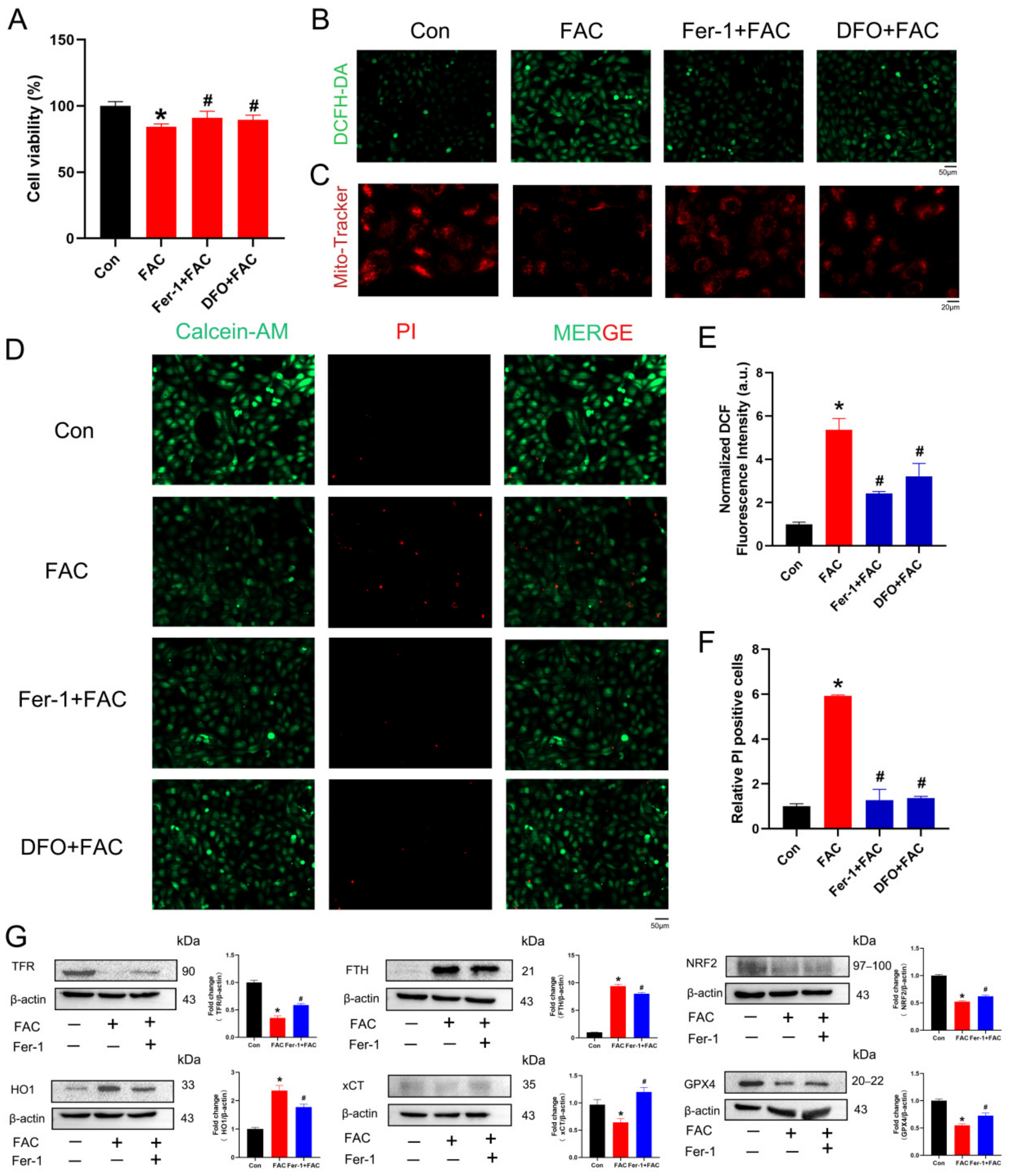

Figure S1. FAC-induced ferroptosis was reversed using ferrostatin-1in HK-2 cells.

(A)HK-2 cells were treated with the iron chelator DFO (50 μM), the ferroptosis inhibitor ferrostatin-1 (Fer-1, 1 μM) and cell viability was assayed using CCK-8 (*n* = 5). (B、E) Increase in cellular ROS levels and quantitative analysis graph in HK-2 cells following

exposure, with the use of Fer-1 and DFO (*n* = 3). (C) Representative microscopic images of HK-2 cells stained with Mito-Tracker Red. (D、F) Calcein-AM/PI staining of HK-2 cells and the proportion of PI-positive cells following treatment with Fer-1 and DFO (*n* = 3). (G) Differential expression of ferroptotic proteins in HK-2 cells was reversed using Fer-1 (*n* = 3). Data are presented as mean ± SEM. $^{*}P < 0.05$ vs. the control group. $^{\#}P < 0.05$ vs. the FAC group.

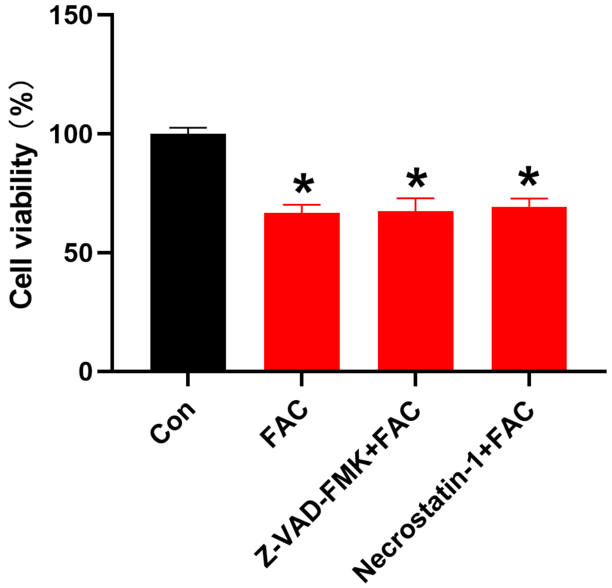

Figure S2. Effects of apoptosis and necroptosis inhibitors on FAC-induced HK-2 cell death.

HK-2 cells were treated with FAC in the presence or absence of the apoptosis inhibitor Z-VAD-FMK (20 μM) or the necroptosis inhibitor Necrostatin-1 (100 μM). Cell viability was measured using the CCK-8 assay (*n*=5). Data are presented as mean ± SEM. **P* <0.05 vs. the control group. #*P* <0.05 vs. the FAC group.

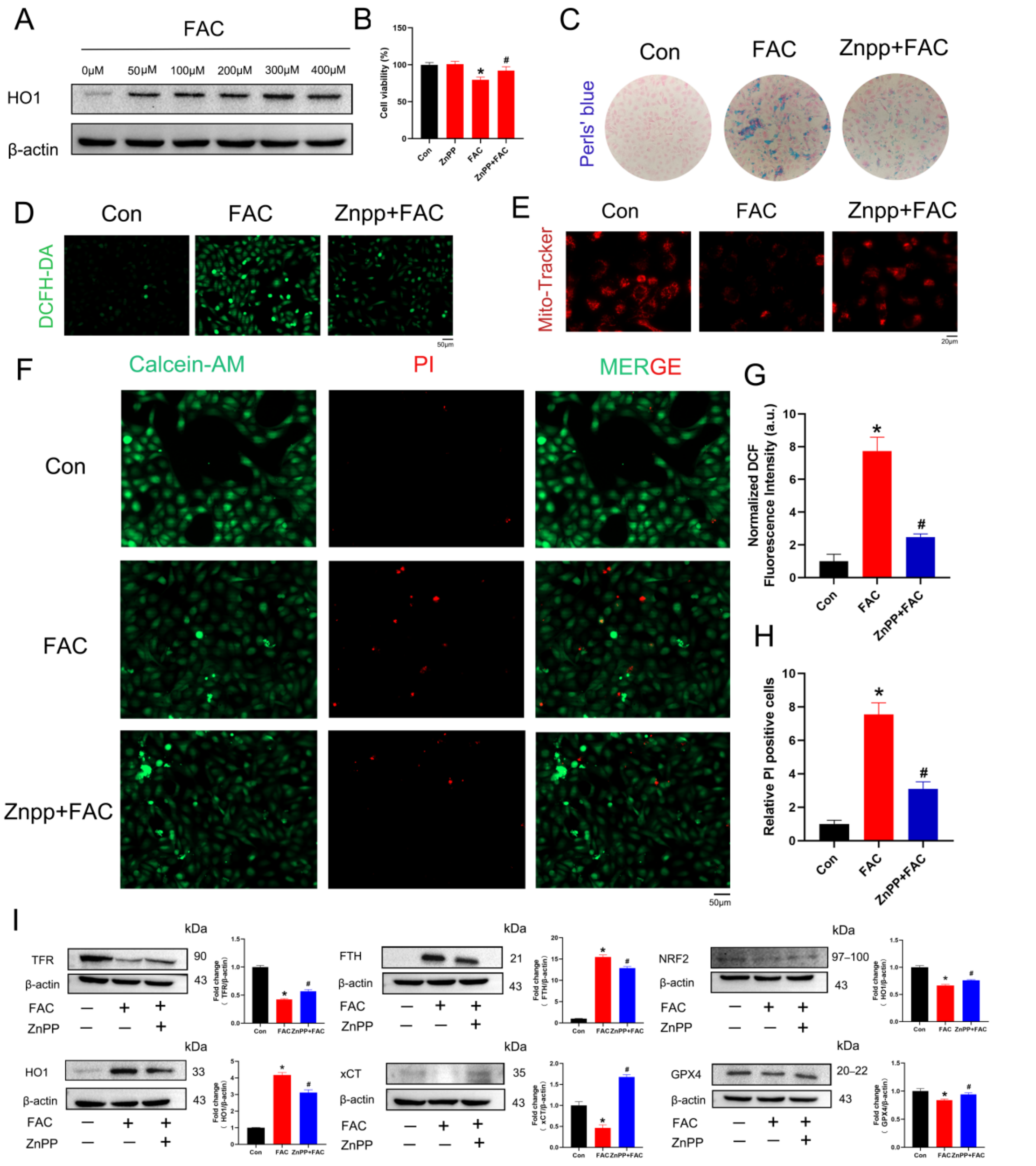

Figure S3. FAC induced ferroptosis via HO1 in HK-2 cells.

(A) Western blotting analysis showed that FAC upregulated the expression of HO1 in a dose-dependent manner in HK-2 cells. (B) CCK-8 assay showed that FAC-induced cell death was mitigated in the presence of ZnPP (1μM) (*n* = 5). (C) FAC increased intracellular iron in HK-2 cells and pretreatment with ZnPP mitigated. (D、G) Changes in ROS fluorescence intensity and quantitative analysis in HK-2 cells treated with ZnPP (*n* =

3). (E) Representative microscopic images of HK-2 cells stained with Mito-Tracker Red. (F、H) The effect of ZnPP on FAC-induced cell death was analyzed by Calcein-AM/PI staining (*n* = 3). (I) Differential expression of ferroptotic proteins in HK-2 cells was reversed using ZnPP (*n* = 3). Data are presented as mean ± SEM. *P <0.05 vs. the control group. $^{\#}P$ <0.05 vs. the FAC group.

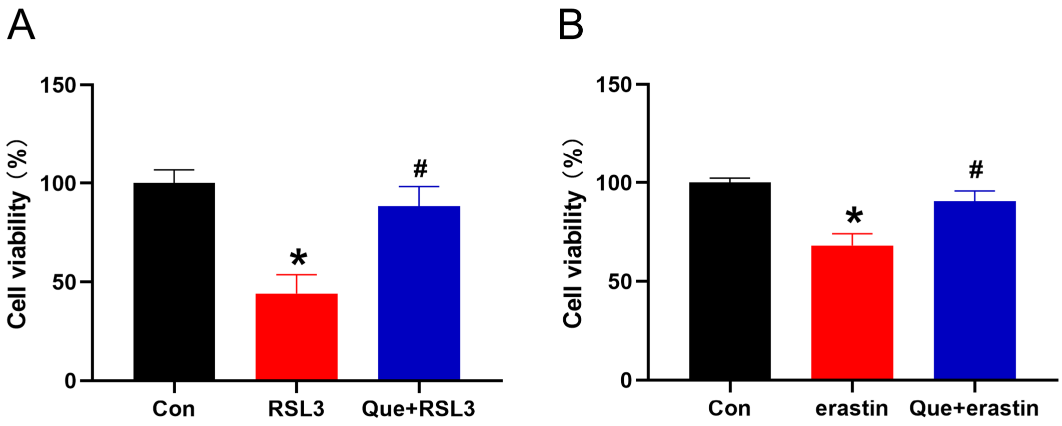

Figure S4. Que alleviates RSL3- and Erastin-induced ferroptotic cell death in HK-2 cells.

(A-B) HK-2 cells were treated with the ferroptosis inducers RSL3 or Erastin in the presence or absence of Que (10 μM). Cell viability was assessed using the CCK-8 assay after treatment. Both RSL3 and erastin significantly reduced HK-2 cell viability, whereas Que treatment partially restored cell viability under these conditions (*n*=5). Data are presented as mean ± SEM. **P* <0.05 vs. the control group. #*P* <0.05 vs. the Que group.
